# Insulin Resistance and Pellino-1 Mediated Decrease in the Activities of Vasodilator Signaling Contributes to Sunitinib-Induced Hypertension

**DOI:** 10.3389/fphar.2021.617165

**Published:** 2021-03-25

**Authors:** Yang Liu, Liang-Liang Tang, Chen Liang, Ming-Ming Wu, Zhi-Ren Zhang

**Affiliations:** ^1^ Departments of Cardiology and Clinical Pharmacy, Harbin Medical University Cancer Hospital, Institute of Metabolic Disease, Heilongjiang Academy of Medical Science, Heilongjiang key laboratory for Metabolic disorder and cancer related cardiovascular diseases, and Key Laboratories of Education Ministry for Myocardial Ischemia Mechanism and Treatment, Harbin, China; ^2^ NHC Key Laboratory of Cell Transplantation, Harbin Medical University, Harbin, China

**Keywords:** sunitinib, hypertension, insulin resistance, endothelial cell, vasodilatation

## Abstract

Antiangiogenic tyrosine kinases inhibitors induce hypertension, which may increase the incidents of cardiovascular complications and limit their use. However, the mechanisms by which usage of TKIs results in hypertension have not been fully understood. Here, we report the potential mechanisms of how sunitinib, a widely used TKI, induces hypertension. Male SD rats were randomly divided into control group and sunitinib-administrated group. We show that sunitinib administration for seven days caused a significant increase in artery blood pressure, along with glycerolipid metabolism abnormalities including decreased food intake and low body weight, hypoglycemia, hyperinsulinemia. Sunitinib administration also resulted in a significant increase in the levels of insulin autoantibody (IAA), cyclic adenosine monophosphate and free fatty acid in serum; whereas, sunitinib administration had no effects on serum glucagon levels. Sunitinib led to the decreased insulin sensitivity as determined by insulin tolerance test (ITT) and glucose tolerance test (GTT), reflecting insulin resistance occurred in sunitinib-treated rats. The results obtained from wire myograph assay in the mesenteric arteries show that endothelium-dependent relaxation, but not endothelium-independent relaxation, was impaired by sunitinib. Furthermore, western blot analysis revealed that the expressions levels of phosphorylated IRS-1, Pellino-1, AKT and eNOS were significantly attenuated by sunitinib in rat mesenteric artery tissues and in the sunitinib-treated primary cultured mesenteric artery endothelial cells. The levels of serum and endothelium-derived nitric oxide were also significantly decreased by sunitinib. Moreover, sunitinib-induced decrease in the expression levels of phosphorylated AKT and eNOS was further reduced by knocking down of Pellino-1 in MAECs. Our results suggest that sunitinib causes vascular dysfunction and hypertension, which are associated with insulin resistance- and Pellino-1-mediated inhibition of AKT/eNOS/NO signaling. Our results may provide a rational for preventing and/or treating sunitinib-induced endothelial dysfunction and hypertension.

## Introduction

Sunitinib, as a member of small molecular tyrosine kinase inhibitor family, has been widely used to treat a variety types of cancer including gastrointestinal stromal tumors with secondary resistance to imatinib, advanced pancreatic neuroendocrine tumor and inoperable renal cell carcinoma (Demetri et al., 2006; Motzer et al., 2007; Raymond et al., 2011). Previous studies have suggested that sunitinib not only inhibits vascular endothelial growth factor (VEGF) receptors, but also targets other related receptor tyrosine kinases including platelet-derived growth factor receptors, stem cell factor receptor, fms-related tyrosine kinase 3, colony stimulating factor 1 receptor, and glial cell line-derived neurotrophic factor receptor (Aita et al., 2012). Beyond the clinical benefits of sunitinib, the elevated blood pressure (BP) is a substantial side-effect with an overall incidence of 21.6% among the patients treated with sunitinib (Li et al., 2015). Furthermore, it has been reported that sunitinib often causes severe hypertension in the patients with a history of high BP (Bamias et al., 2011). Despite the occurrence of sunitinib induced hypertension has considered as a sign of drug response or expecting a longer tumor progression-free survival, proper management should be conducted to prevent cancer patients from developing severe cardiovascular complications secondary to sunitinib-induced hypertension (Donskov et al., 2015). Therefore, revealing the underlying mechanisms of sunitinib-induced hypertension is an important issue.

Regarding the pathogenesis of sunitinib-induced hypertension, the most studies have focused on the plausible factors involving oxidative stress, renal dysfunction, reduction of nitric oxide (NO) levels and endothelin system over-activation. However, the precise contributions of these factors to the pathogenesis of sunitinib-induced hypertension remain to be further elucidated (Sourdon et al., 2017; Witte et al., 2018). Currently, it is well accepted that sunitinib disturbs the balance between the vasodilator NO and ET-1, favoring the increased activity of ET-1 (León-Mateos et al., 2015). The reduced NO production/release in the arterioles and other resistance vessels is considered as the cornerstone mechanism of endothelial impairment and hypertension in sunitinib-treated patients (Izzedine et al., 2009; Thijs et al., 2015).

A recent clinical study has demonstrated that early use of sunitinib resulted in the decreased insulin clearance and insulin sensitivity in patients with metastatic renal cell carcinoma (Thijs et al., 2016). This study provided a clue that there might be a relationship between insulin resistance (IR) and sunitinib-induced hypertension. IR has been recognized as an independent risk factor of hypertension, and insulin mediated-activation of endothelial IRS-1/PI3K/AKT/eNOS pathway is unique biological action for regulating vasodilation and arterial pressure (Andreozzi et al., 2004). Attenuation of endothelial insulin signaling in IR condition leads to a reduction of NO generation and endothelial damage, thereby promoting the development of hypertension (EI Assar et al., 2015; León-Mateos et al., 2015; Lin et al., 2018).

In addition, VEGFR2/Flk-Pellino-1/AKT/eNOS signaling are also characterized as the potential regulators of angiogenesis and NO production in cardiomyocytes. In this study the authors demonstrated that the expression levels of Pellino-1 and the activities of AKT/eNOS in cardiomyocytes were attenuated by knocking down of VEGFR2, suggesting that Pellino-1 plays an important role in angiogenesis and cardiac repair downstream to VEGF/Flk (Thirunavukkarasu et al., 2018). Given the facts that sunitinib has multiple pharmacological targets and that sunitinib treatment affects insulin clearance and insulin sensitivity, we therefore hypothesized that sunitinib-induced hypertension is associated with insulin resistance, insulin signaling and Pellino-1/AKT/eNOS pathways. We used a variety of approaches to test this hypothesis.

## Materials and Methods

### Materials

Several studies suggest that estrogen affects insulin signaling, vascular function, and even lipoprotein metabolism, and these physiological effects of estrogen may generate bias to our experimental results (Knopp et al., 1994; Lee et al., 1999; Ceballos et al., 2000). Therefore, we selected male Sprague-Dawley (SD) rats (weighing 220–280 g) supplied by animal center located at the Second Affiliated Hospital of Harbin Medical University. All animal handling procedures were approved by the Harbin Medical University Animal Supervision Committee.

Sunitinib L-malate (E129728-1) was purchased from Aladdin Industrial Corporation (Shanghai, China). Antibodies employed were as follows: IRS-1 (#2382S), AKT (#2920S) and phospho-Ser473 AKT (#4060S) were purchased from Cell Signaling Technology (Danvers, MA, United States); phospho-Tyr 612 IRS-1 (44-816G) was purchased from Thermo Fisher Scientific Inc. (Rockford, IL, United States); eNOS (ab199956) and Pellino-1 (ab199336) were purchased from Abcam Inc. (Cambridge, MA, United States); phospho-Ser 1177 eNOS (sc-81510) was purchased from Santa Cruz Biotechnology Inc. (Dallas, TX, United States); anti-β-actin antibody (bsm-33036M) was purchased from Bioss Biotechnology Inc. (Peking, China). Mouse/Rabbit HRP-conjugated secondary antibodies (P/N: 926-32210, 926-32211, 926-68071, 926-68070) were obtained from LI-COR Biosciences (Lincoln, NEB, United States). ELISA assay kits for serum cAMP, insulin (INS) and GC detection were purchased from Cloud-Clone Diagnostic Reagents Institute (Wuhan, Hubei, China). Insulin autonomic antibody assay kit was from CUSABIO BIOTECH Co. Ltd (Wuhan, Hubei, China). Nitrate/Nitrite assay kit was purchased from Beyotime Biotechnology (Shanghai, China). FFA chemistry kit was purchased from Nanjing Jiancheng Bioengineering Institute (Nanjing, Jiangsu, China). Recombinant human insulin was purchased from Wanbang Pharmaceutical Co., Ltd., (Jiangsu, China). Dulbecco's modified eagle medium (DMEM; 10013-cvrc) used for MAECs culture were purchased from Corning Incorporated (United States). Penicillin and streptomycin (C0222) were from Beyotime Biotechnology (Shanghai, China) and FBS was from Beijing Sage Creation Science Co. Ltd. (Peking, China).

### The Dose of Sunitinib Used *In Vivo* and *In Vitro* Experiments

In the *in vivo* studies, sunitinib L-malate 27.5 mg/kg per day was used in rats and 1 μM sunitinib was used in the *in vitro* experiments. The usage of these doses was according to the previous observations (Kerkela et al., 2009; Farsaci et al., 2012; Fiorio Pla et al., 2014; Jacob et al., 2016); where the IC_50_ values of sunitinib was determined by activity against various protein kinases and pro-angiogenic functions in normal endothelial cells.

### Isolation and Culture of Rat MAECs

Methods for isolation, culture and identification of rat mesenteric artery endothelial cells (MAECs) were described in our previous studies (Liu et al., 2015; Wang et al., 2018). Briefly, male SD rats were sacrificed and mesentery containing arteries were isolated, then the oozing blood from arteries was perfused and washed out with sterile and chilled physiological saline solution (PSS) containing (in mM): 137 NaCl, 5.4 KCl, 0.05 CaCl_2_, 0.4 KH_2_PO_4_, 0.4 Na_2_HPO_4_, 4.4 NaHCO_3_ and 10 HEPES (pH 7.4 with HCl). We cut each mesenteric artery of rats into four parts, and immediately put the segments of artery into the pre-prepared collagenase I with a proportion of 0.2 mg ml^−1^. One hour after digestion at 37°C, the process was stopped by cell-culture medium, the filtered cell-culture medium was collected and the MAECs was extracted by centrifugal force at 1200 × g for 5 min. After removing the supernatant, MAECs were purified repeatedly from fibroblasts and blood system cells using differential adhesion method. Final isolated MAECs were grown in DMEM supplemented with 20% FBS and 1% penicillin/streptomycin at 37°C in 5% CO_2_ for 5 days. Until cells reached confluence, sunitinib (1 μM) was added and the cells were incubated for 30 min, then the cell-culture medium was removed, the cells attached to the bottom of the dishes were washed with sterile PSS, and lysed at 4 C for 20 min in RIPA buffer (Fiorio Pla et al., 2014). The extracted total protein is prepared for western blotting.

### Animals and Treatments

Male SD rats were housed in an isolated room with controlled environment, as a 12 h light/12 h dark cycle (temperature: 22–24°C). The rats had free access to food and water. Before the further experiments, rats were allowed to adapt to their living environment for a week. After measuring the baseline parameters including BP, rats were randomly assigned into two groups: the control group fed with a standard diet and drinking water and the sunitinib-administrated group fed a standard diet and drinking water supplemented with sunitinib L-malate 27.5 mg/kg per day (Lankhorst et al., 2014). In order to determine exogenous insulin stimulated activation of insulin signaling pathway in rat mesenteric arteries, the rats were fasted for 12 h followed by intraperitoneal injection of 5 μ kg^−1^ BW of recombinant human insulin. Ten minutes after injection, the total protein was extracted and western blot was performed.

### Measurement of Blood Pressure and Glucose

Before and 7 days after sunitinib administration, the systolic arterial blood pressure (SABP) was measured in conscious rats with tail-cuff method (BP 98A, Softon, Tokyo, Japan), as described previously (Feng et al., 2008). Food intake and body weight of rats were monitored daily up to the day of execution. Randomized blood glucose (RBG) levels were detected via a tail vein puncture using a glucometer and corresponding glucose strips (Johnson, United Kingdom) at days 3, 5, 7 after sunitinib administration. Rats were fasted for 12 h before detecting fasting blood glucose (FBG) levels.

### Glucose and Insulin Tolerance Tests

Glucose tolerance tests (GTT) and insulin tolerance tests (ITT) were performed at the end of the administration. Referring to the description of previous studies (Jia et al., 2014; Yu et al., 2018), rats were fasted for 5 h; baseline glucose levels were measured before the tests, then the blood glucose levels were measured at the time points of 15, 30, 60, and 120 min after intraperitoneal injection of 1.2 mg g^−1^ BW glucose. For ITT, blood glucose levels of 5 h fasted rats were determined at the same time points as GTT after intraperitoneal injection of recombinant human insulin at 1.5 mU g^−1^ BW.

### Measurements of Serum Parameters

Blood samples were collected from abdominal vein of sacrificed rats and kept in a separate gel coagulation promoting vacuum tube. Following centrifugation at 2,000 g for 20 min, serum was removed and frozen at −80°C for subsequent analyses. The levels of serum cAMP, insulin, glucagon, and IAA were detected using ELISA kits (Cloud-Clone Diagnostic Reagents Institute, Wuhan). For IAA detection, the serum samples were pre-diluted 2,000-fold to improve the accuracy of detection. FFA concentrations were analyzed using biochemical method as previously described (Wang et al., 2006). All experimental procedures were conducted according to the manufacturer's instructions and the data were analyzed in multiple duplicates.

### Measurement of NO Production in Serum and the Endothelial Cells of Mesenteric Artery

As the half-life of NO is very short, total NO production in serum and endothelial cells were calculated by measuring the concentration of nitrate and nitrite, the stable metabolite of NO, with Griess reagent using the NO assay kit (Beyotime Company, Haimen, China) according to the manufacturer’s instructions and our previous study (Liu et al., 2015).

### Wire Myograph Detection

Artery relaxations were measured using an isometric myograph (Danish Myo Technology, Aarhus, Denmark), as previously described (Yang et al., 2010; Liu et al., 2015; Wang et al., 2018). Briefly, second-order mesenteric resistance arteries (diameter 100–200 μm, length 1.8–2 mm) were mounted in a Mulvany wire myograph with PSS and oxygenated with 95% O_2_ and 5% CO_2_ at 37°C. Artery rings were pre-contracted with phenylephrine (10 μM). Endothelium-dependent and -independent relaxations were determined by measuring a cumulative dose–response curve to acetylcholine (ACh: 0.1 nM to 100 μM) and nitroglycerin (NTG: 0.1 nM to 10 μM), respectively.

### Western Blotting

None-insulin and insulin stimulated IRS-1/AKT/eNOS activation in rat arteries were tested with western blot, as described previously (Andreozzi et al., 2004; Zheng et al., 2016; Wu et al., 2019). Accordingly, rats were fasted for 12 h at the 7^th^ day after sunitinib administration. Physiological saline or 5 μkg^−1^ BW of recombinant human insulin was injected intraperitoneally. 10 min after insulin injection, isolated mesenteric vascular tissue were quickly removed, then stored at −80°C. Protein from cultured endothelial cells (mentioned in the section of isolation and culture of rat MAECs) and mesenteric arteries were extracted respectively using lysing buffer (Beyotime, Shanghai, China) containing protease inhibitors (P2714, Sigma) and phosphatase inhibitor (PI-78420, Thermo Scientific). Protein concentration was determined by using BCA protein assay kit (Peking Applygen Technologies Inc., China). Proteins were subjected to SDS-PAGE, transferred to nitrocellulose filter membrane, blocked with 5% no-fat milk or 5% bovine serum albumin for 1 h at room temperature (22–24°C). Blots were incubated overnight at 4°C with primary antibodies including anti-IRS-1 (diluted at 1:1,000), anti-phospho-Tyr612 IRS-1 (diluted at 1:1,000), anti-AKT (diluted at 1:1,000), anti-phospho-Ser473AKT (diluted at 1:1,000), anti-eNOS (diluted at 1:1,000), anti-phospho-Ser1177 eNOS (diluted at 1:500), anti-Pellino-1 (diluted at 1:1,000) and anti-*β*-actin (diluted at 1:1,000). Following the incubation with primary antibodies, the membranes were incubation with the Mouse/Rabbit HRP-conjugated secondary antibodies (diluted at 1:10,000) at room temperature (22–24°C) for 1 h; then the blots were visualized using chemiluminescent peroxidase substrate and then quantified with Image Studio (Version 2.1).

### Knocking Down Pellino-1 Expression in MAECs Using shRNA

The isolated MAECs were cultured for 5 days and then were respectively transfected with the three different lentivirus-packed rat shRNAs against Pellino-1 and scramble shRNA (GenePharma, Shanghai, China). The sequences of sh-RNAs are as follows: Peli1-a, 5′-CCT​GGA​ATA​TGG​AGA​GAG​ATA-3′; Peli1-b, 5′-GAC​GGC​AAA​GAT​CGT​GAA​TGT-3′; Peli1-c, 5′-ACC​AGC​ATA​GCA​TAT​CGT​ATA-3′; the sequence of scramble shRNAs is: 5′-TTCTCCGAACGTGTCACGT-3′.The knocking down efficiency of Pellino-1 were determined by western blots.

### Reagents and Chemicals

Unless otherwise noticed, all reagents and chemicals were purchased form Sigma Aldrich (Sigma-Aldrich, United States).

### Statistical Analyses

Statistical analyses were performed using SPSS software package (Version 20.0). Quantitative data were expressed as means and standard error of the mean (Mean ± SEM). Data were analyzed using one-way or two-way ANOVA procedure, followed by turkey test, paired or independent sample *t* tests. A *p* value <0.05 was considered as statistical significance.

## Results

### Sunitinib Administration Led to an Increase in BP and Impairment of Endothelial-Dependent Relaxation in SD Rats

**FIGURE 1:**
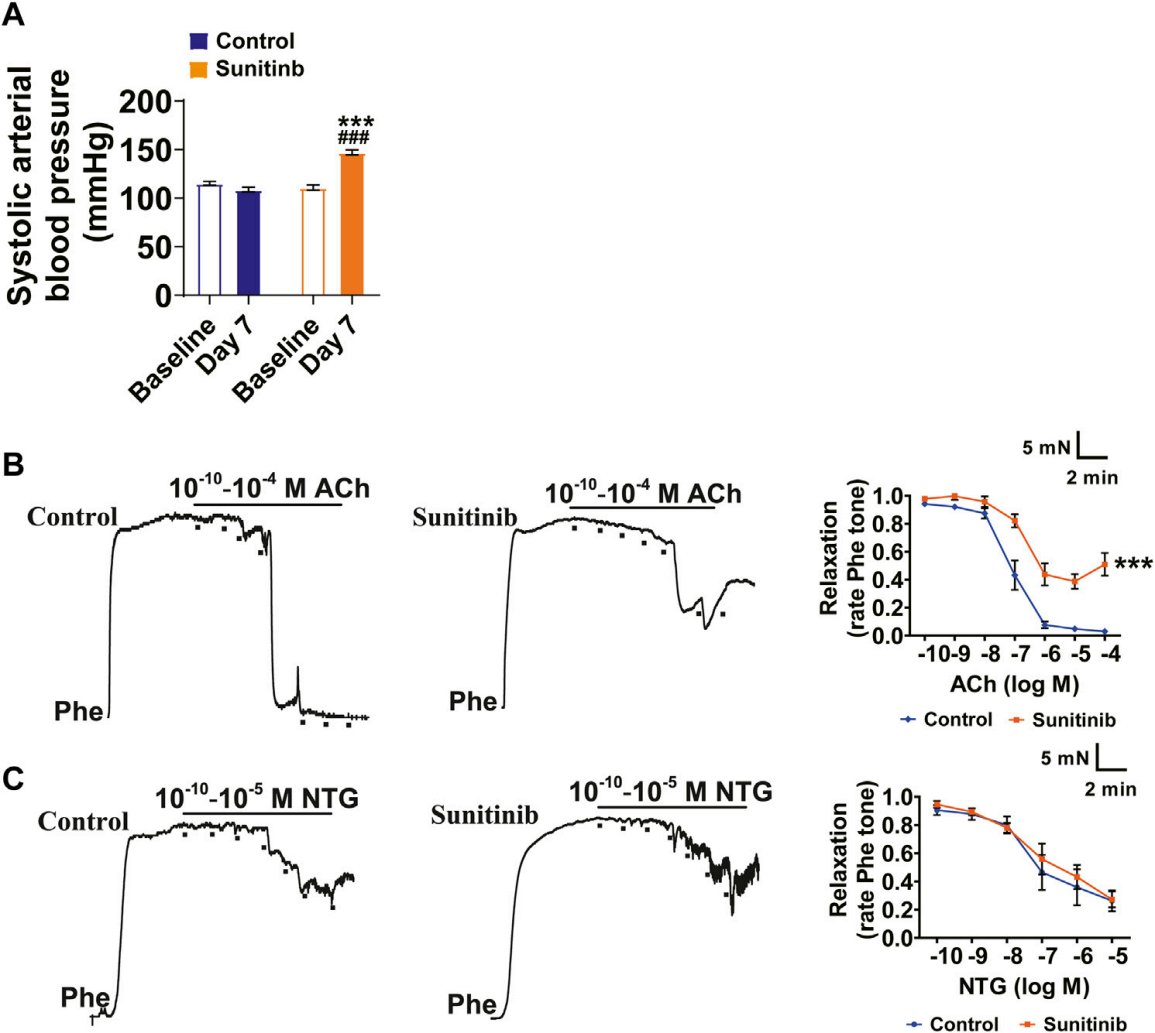
Sunitinib administration led to an increase in sBP and impairment of endothelial-dependent relaxation in SD Rats. **(A)** Systolic arterial blood pressure of rats measured at baseline and 7 days after sunitinib treatment (^***^ *p* < 0.001 vs. control rats at day 7; ^###^ *p* < 0.001 vs. baseline BP of sunitinib-administrated rats; *n* = 18 rats/group). **(B)** Representative raw data and summarized plots of ACh-induced relaxation in Phe-precontracted mesenteric artery rings isolated from control rats or rats treated with sunitinib for 7 days (^***^ *p* < 0.001 vs. control; *n* = 6 rats/group). **(C)** Representative traces and summarized plots of NTG-induced relaxation in Phe-precontracted mesenteric artery rings isolated from control rats or sunitinib-treated rats for 7 days (*p* > 0.05 vs. control; *n* = 6 rats/group).

Sunitinib causes hypertension (Kappers et al., 2010; Bamias et al., 2011; Li et al., 2015); however, the mechanisms by which sunitinib induces hypertension remain to be elucidated. To this end, we used rats as an experimental model to decipher the potential mechanisms of sunitinib-induced hypertension. We measured systolic arterial blood pressure (sBP) in the conscious rats prior to sunitinib administration (defined as the baseline sBP of rats) and seven days after application of sunitinib. Our results show that there were no statistical differences in baseline sBP between the control and sunitinib-treated groups. However, after 7 days of sunitinib administration, the mean sBP of rats was significantly elevated as compared with either their own baseline sBP or with sBP of control rats (Figure 1A).

Endothelium-dependent vasodilation plays an important role in vascular homeostasis and is associated with pathological stimuli-induced increase in BP (Yang et al., 2010; Liu et al., 2015; Wang et al., 2018). Therefore, we examined whether sunitinib impairs vascular relaxation using the second-order mesenteric resistance arteries. The results manifested that maximum ACh-induced vasorelaxation in the mesenteric arteries was impaired by sunitinib compared with the control (Figure 1B). However, sunitinib did not affect the NTG-induced vasorelaxation (Figure 1C).

### Sunitinib Administration Led to Metabolic Disorders and a Decrease in Insulin Sensitivity in SD Rats

**FIGURE 2:**
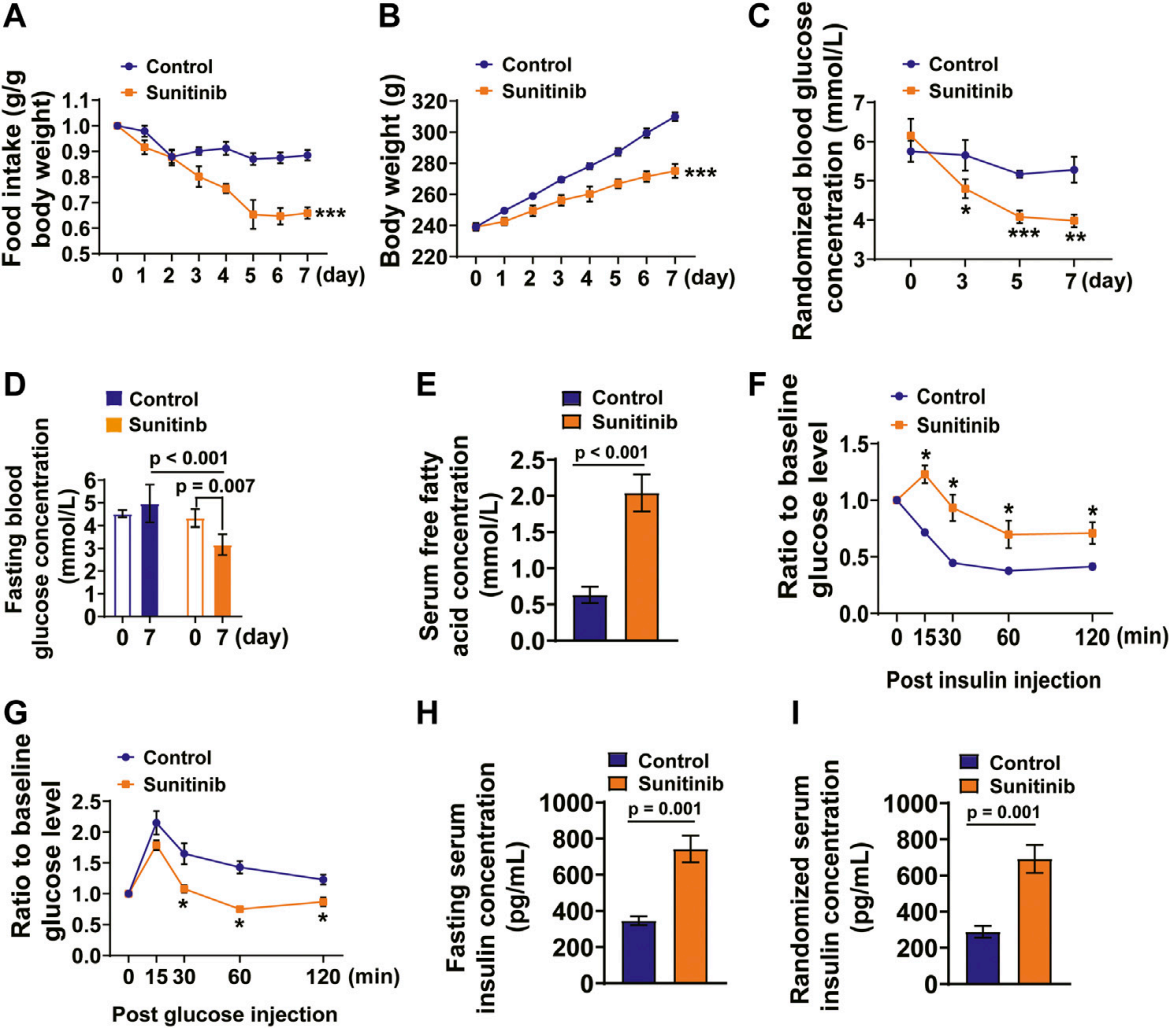
Sunitinib administration led to metabolism disorders and insulin resistance in SD rats. **(A)** Average daily food intake was dramatically decreased by sunitinib administration (^***^ *p* < 0.001 vs. control; *n* = 6 rats/group). **(B)** Average daily body weight was significantly decreased by sunitinib administration (^***^ *p* < 0.001 vs. control; *n* = 6 rats/group). **(C)** RBG concentration detected on day 3, 5, 7 in control rats or sunitinib-treated rats (*p < 0.05 vs. control; **p < 0.01 vs. control, ***p < 0.001 vs. control; *n* = 6 rats/group). **(D)** FBG concentration measured on day 7 in control rats or sunitinib-treated rats (*p* = 0.007 vs. baseline; *p* < 0.001 vs. control at day 7; *n* = 6 rats/group). **(E)** Serum FFA concentration (*p* < 0.001 vs. control; *n* = 9 rats/group) detected on day 7 in control rats or the rats treated with sunitinib. **(F)** On the 7^th^ day, ITT assay (intraperitoneal injection of recombinant human insulin 1.5 mUg^−1^ BW) performed in control and sunitinib-treated rats that were fasted for 5 h; the statistical results presented as follows: were present tested value obtained from each rat, measured at the indicated time points, was normalized to its own baseline value hours (^*^ *p* < 0.05 vs. control; *n* = 6 rats/group) **(G)** Glucose tolerance test (intraperitoneal injection of glucose 1.2 mg g^−1^ BW) performed on day 7 in control and sunitinib-treated rats were fasted for 5 h; the statistical results presented as follows: tested value obtained from each rat, measured at the indicated time points, was normalized to its own baseline value (^*^ *p* < 0.05 vs. control; *n* = 6 rats/group). **(H)** Fasting serum insulin concentration was detected on day 7 in control and sunitinib-treated rats (*p* = 0.001 vs. control; *n* = 7 rats/group). **(I)** Randomized serum insulin concentration detected on day 7 in control and sunitinib-treated rats (*p* = 0.001 vs. control; *n* = 7 rats/group).

Sunitinib treatment is often accompanied by gastrointestinal toxicity, low body weight and hypoglycemia (Motzer et al., 2007; Bhojani et al., 2008; Steeghs et al., 2008; French et al., 2010). We reasoned that sunitinib-induced hypoglycemia may result in free fatty acid (FFA) release. Therefore, we measured the parameters, reflecting the metabolic and nutritional status of the rats, before (baseline value) and seven days post sunitinib administration. Our data show that the average daily food intake (as determined by normalizing the values of sunitinib treatment to the baseline values) was significantly reduced by sunitinib compared with control rats (Figure 2A). The increase in body weight was significantly faster in control rats than in rats treated with sunitinib (Figure 2B).

We then examined the concentrations of randomized blood glucose (RBG) and fasting blood glucose (FBG) in both the control and sunitinib-administrated rats. The data show that there was no significant difference in the baseline RBG levels between two groups; moreover, it appears that there were no significant differences between the RBG levels measured on days 3, 5, and 7and the baseline RBG levels of control group (the black line; Figure 2C). However, sunitinib administration led to a significant decrease in RBG levels as early as on day 3, and declined further on days 5 and 7 (the red line; Figure 2C). Moreover, sunitinib administration for 7 days resulted in the much lower FBG levels compared to either their own baseline levels or to the control (Figure 2D). In contrast, application of sunitinib for 7 days led to a significant increase in the concentration of FFA (∼3.2-fold) compared with the control rats (Figure 2E).

Previous clinical study has demonstrated that usage of sunitinib led to the decreased indices of insulin sensitivity and to an increased insulin levels in the patients with metastatic renal cell carcinoma (Thijs et al., 2016). Therefore, we performed insulin tolerance test (ITT) and glucose tolerance test (GTT), which could evaluate the insulin sensitivity, glucose utilization and glucose-dependent insulin secretion (Jia et al., 2014; Yu et al., 2018). The glucose levels were respectively measured before (baseline levels) and post injections of insulin and glucose (tested values). We analyzed and presented the results of ITT and GTT as follows: the tested value obtained from each rat, measured at the indicated time points, was normalized to its own baseline value, which represents injection of insulin- or glucose-induced alteration of the serum glucose level. The results show that post insulin injection the serum glucose levels were significantly reduced in the control rats compared to that in sunitinib treated rats, suggesting that sunitinib induced impairment of insulin sensitivity (Figure 2F). Moreover, 30 min after glucose injection the serum glucose levels of sunitinib treated rats were significantly lower than that of control rats (Figure 2G).

### Sunitinib Administration Increased Serum Insulin, IAA and cAMP Levels and Had No Effects on Serum Glucagon Levels

We reasoned that the results of GTT obtained from sunitinib-treated rats might be associated with an enhanced glucose-induced insulin secretion and the impaired renal insulin clearance, which may lead to spontaneous hypoglycemia (Malik et al., 2016; Mesmar et al., 2020). Therefore, we detected the levels of serum insulin and glucagon under both the fasting and feeding conditions, respectively. The results showed that serum insulin levels, under both the fasting and feeding conditions, were significantly increased by sunitinib compared with the control rats (Figures 2H,I).

**FIGURE 3:**
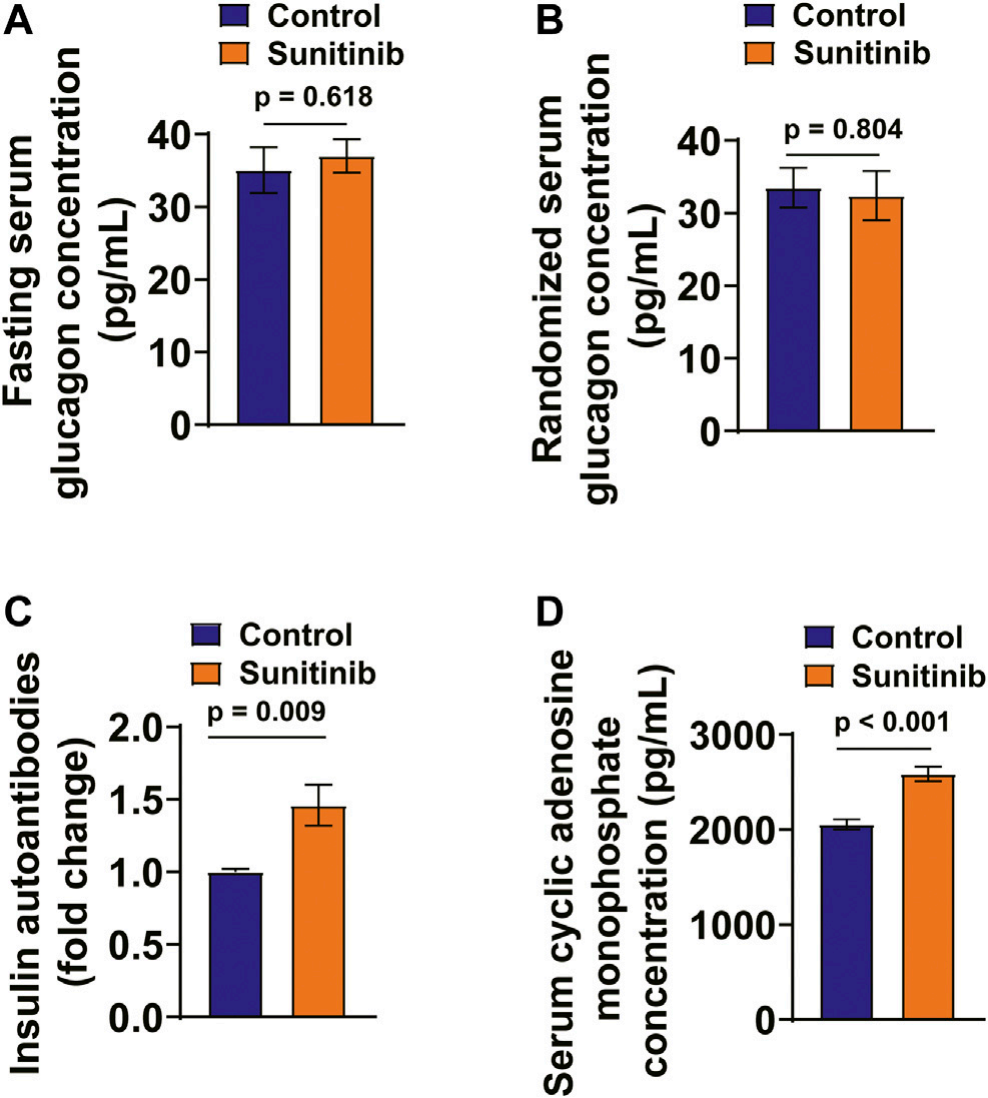
Sunitinib Administration Increased Serum IAA and cAMP Levels and Had No Effects on Serum Glucagon Levels. **(A)** Fasting serum glucagon concentration measured on day 7 in control and sunitinib-treated rats (*p* = 0.618 vs. control; *n* = 15 for control; *n* = 25 for sunitinib-treated group). **(B)** Randomized serum glucagon concentration measured on day 7 in control and sunitinib-treated rats (*p* = 0.804 vs. control; *n* = 10 rats/group). **(C)** The titer of serum insulin autoantibody (IAA) detected in control and sunitinib-treated rats (*p* = 0.009 vs. control; *n* = 7 rats/group). **(D)** Serum cAMP levels of control and sunitinib-treated rats detected on day 7 (*p* < 0.001 vs. control; *n* = 8 rats/group).

The serum glucagon levels between the two groups were virtually the same (Figures 3A,B). Furthermore, the occurrence of IAA is a rare event, which is associated with hyperinsulinemia and may cause hypoglycemia (Ismail, 2016). Therefore, we examined the effect of sunitinib on the levels of serum IAA with an ELISA kit. The result showed that the titer of serum IAA was significantly increased by sunitinib (Figure 3C). Furthermore, sunitinib administration significantly increased the levels of serum cAMP in rats (Figure 3D).

### Sunitinib Inhibits IRS-1/AKT/eNOS Pathway in Rat Arteries and MAECs

**FIGURE 4:**
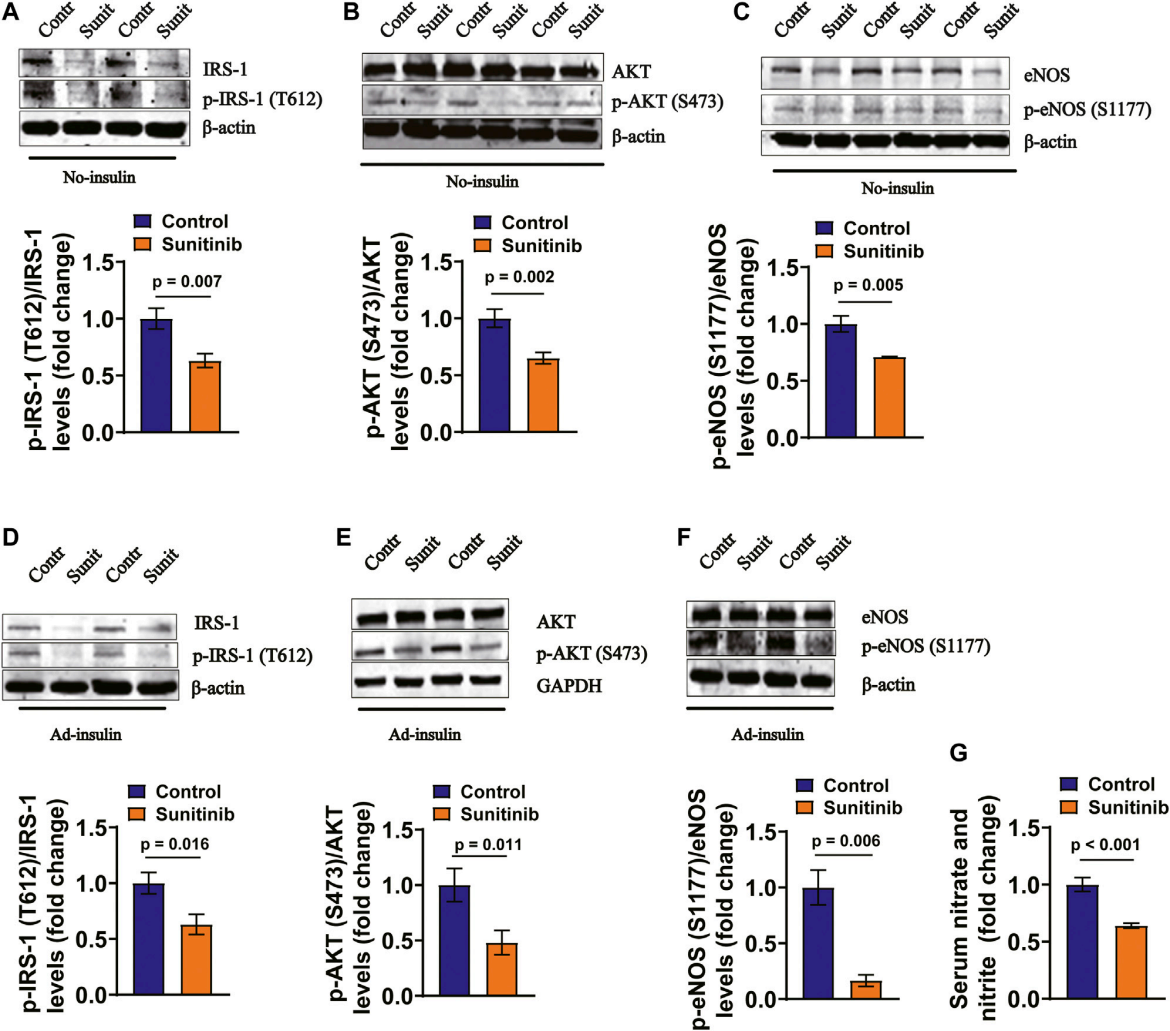
Sunitinib inhibits insulin signaling pathway in mesenteric arteries in rats. **(A)** Representative western blots of total IRS-1 and phospho-Tyr612 IRS-1 in mesenteric artery tissues isolated on day 7 from control and sunitinib-treated rats, without insulin stimulation; bar graph represents phospho-Tyr 612 IRS-1 levels normalized to total IRS-1 levels **(lower panel)** (*p* = 0.007 vs. control; *n* = 6 rats/group). **(B)** Representative western blots of total AKT and phospho-Ser473 AKT in mesenteric artery tissues isolated on day 7 from control and sunitinib-treated rats, without insulin stimulation; bar graph represents phospho-Ser473 AKT levels normalized to total AKT levels **(lower panel)** (*p* = 0.002 vs. control; *n* = 7 rats/group). **(C)** Representative western blots of total eNOS and phospho-Ser1177 eNOS in mesenteric artery tissues isolated on day 7 from control and sunitinib-treated rats, without insulin stimulation; bar graph represents phospho-Ser1177 eNOS levels normalized to total eNOS levels **(lower panel)** (*p* = 0.005 vs. control, *n* = 7 rats/group). **(D)** Representative western blot images of total IRS-1 and phospho-Tyr 612 IRS-1 in mesenteric artery tissues dissected on day 7 from insulin-injection control rats or insulin-injection rats treated with sunitinib; bar graph represents phospho-Tyr612 IRS-1levels normalized to total IRS-1 levels **(lower panel)** (*p* = 0.016 vs. control; *n* = 7 per group). **(E)** Representative westerns of total AKT and phospho-Ser473 AKT in mesenteric artery tissues isolated on day 7 from insulin-injection control rats or insulin-injection rats treated with sunitinib; bar graph represents phospho-Ser473 AKT levels normalized to total AKT levels **(lower panel)** (*p* = 0.011 vs. control; *n* = 12 per group). **(F)** Representative western blots of total eNOS and phospho-Ser 1177 eNOS in mesenteric arteries dissected on day 7 from insulin-injection control rats or insulin-injection rats treated with sunitinib; bar graph represents phospho-Ser1177 eNOS levels normalized to total eNOS levels **(lower panel)** (*p* = 0.006 vs. control; *n* = 6 per group). **(G)** Serum nitrate and nitrite concentrations were respectively measured on day 7 in control rats or rats treated with sunitinib, reflecting NO production in each experimental group (*p* < 0.001 vs. control; *n* = 9 per group).

It has been demonstrated that insulin regulates vessel NO production through IRS-1/PI-3 kinase/AKT/eNOS pathway (Zeng et al., 2000). Therefore, we examined whether the activities of IRS-1/AKT/eNOS were affected by sunitinib in rat arteries. Our data show that application of sunitinib for 7 days led to a significant decrease in the phosphorylation levels of IRS-1/AKT/eNOS in the rat artery tissues, both in the absence of (Figures 4A–C) and in the presence of insulin (Figures 4D–F). Moreover, the serum nitrate and nitrite concentrations were significantly reduced by sunitinib, suggesting that application of sunitinib resulted in a decreased serum NO levels (Figure 4G). These results suggest that sunitinib impairs IRS-1/AKT/eNOS pathway in rat arteries.

**FIGURE 5:**
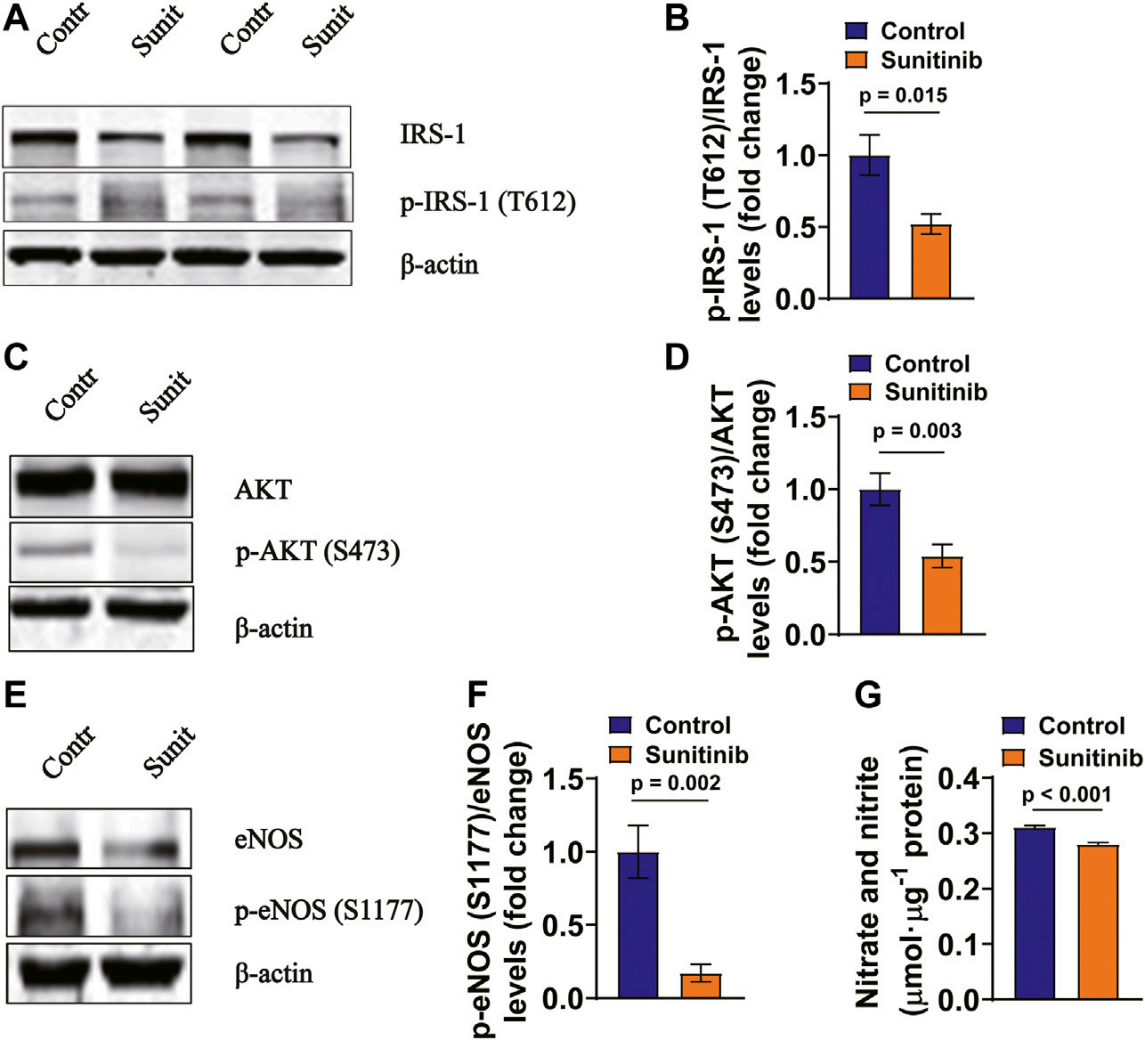
Sunitinib inhibits insulin signaling associated molecules in rat MAECs **(A)** Representative western blots of total IRS-1 and phospho-Tyr612 IRS-1 in control MAECs or in MAECs treated with 1 μM sunitinib for 30 min and bar graph represents phospho-Tyr612 IRS-1 levels normalized to total IRS-1 levels. **(B)** (*p* = 0.015 vs. control; *n* = 6 batches of cells in each group). **(C)** Representative western blot images of total AKT and phospho-Ser473 AKT expression in control MAECs cells or in MAECs treated with 1 μM sunitinib for 30 min and bar graph represents phospho-Ser473 AKT levels normalized to total AKT levels. **(D)** (*p* = 0.003 vs. control; *n* = 7 batches of cells in each group). **(E)** Representative western blot images of total eNOS and phospho-Ser1177 eNOS expression in control MAECs cell or in MAECs treated with 1 μM sunitinib for 30 min and bar graph represents phospho-Ser1177 eNOS levels normalized to total eNOS levels. **(F)** (*p* = 0.002 vs. control, *n* = 7 batches of cells in each group). **(G)** The concentrations of nitrate and nitrite measured in the control MAECs cells or in MAECs treated with 1 μM sunitinib for 30 min, reflecting NO production under indicated experimental condition (*p* < 0.001 vs. control; *n* = 6 per group).

To confirm the results obtained from *in vivo* experiments, primary cultured MAECs were treated with 1 μM sunitinib for 30 min, followed by detecting the phosphorylation levels of IRS-1, AKT and eNOS. We found that phosphorylation levels of IRS-1, AKT and eNOS in MAECs were significantly attenuated by sunitinib as compared to the control (Figures 5A–F). Moreover, concentrations of nitrate and nitrite were significantly decreased by sunitinib, reflecting a reduced endothelium-derived NO production in MAECs (Figure 5G).

### Sunitinib Inhibits Pellino-1 Expression in Rat Arteries and MAECs

**FIGURE 6:**
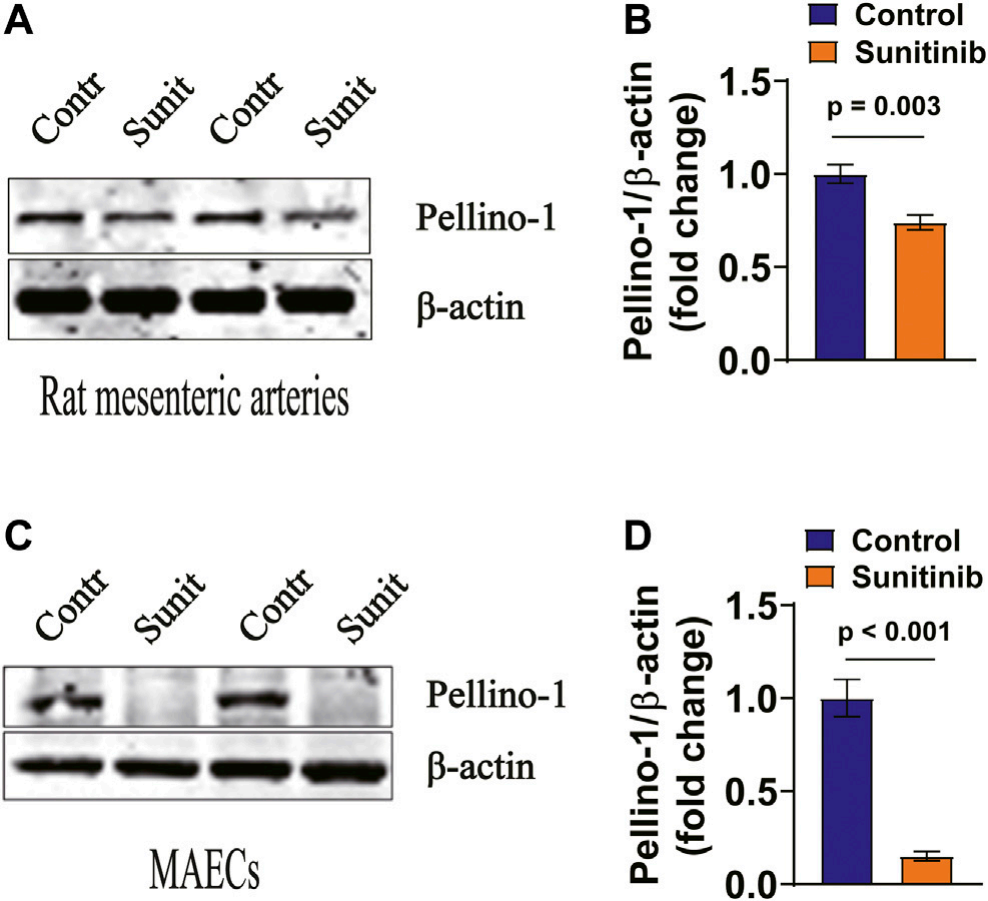
Sunitinib inhibits Pellino-1 expression levels in rat arteries and MAECs. **(A)** Representative western blot images of Pellino-1 expression in rat artery tissues dissected on day 7 from control rats or the rats-treated with sunitinib and bar graph represents Pellino-1 expression normalized to *β*-actin in rat arteries. **(B)** (*p* = 0.003 vs. control; *n* = 6 rats per group). **(C)** Representative western blot images of Pellino-1 expression in control MAECs cells or in MAECs treated with 1 μM sunitinib for 30 min. Statistic bar graph of Pellino-1 expression normalized to *β*-actin in MAECs, summarized from the data shown in. **(D)** (*p* < 0.001 vs. control; *n* = 8 batches of cells in each group).

It appears that acute myocardial infarction-induced reduction of Pellino-1, an important angiogenic molecule under the control of vascular endothelial growth factor (VEGF) receptor 2/Flk-1, leads to reduced activities of AKT and eNOS (Thirunavukkarasu et al., 2018). Therefore, we examined whether sunitinib may also affect AKT and eNOS activities via Pellino-1. Our data show that the expression levels of Pellino-1 was greatly inhibited by sunitinib in both the rat arteries (Figures 6A,B) and MAECs (Figures 6C,D).

**FIGURE 7:**
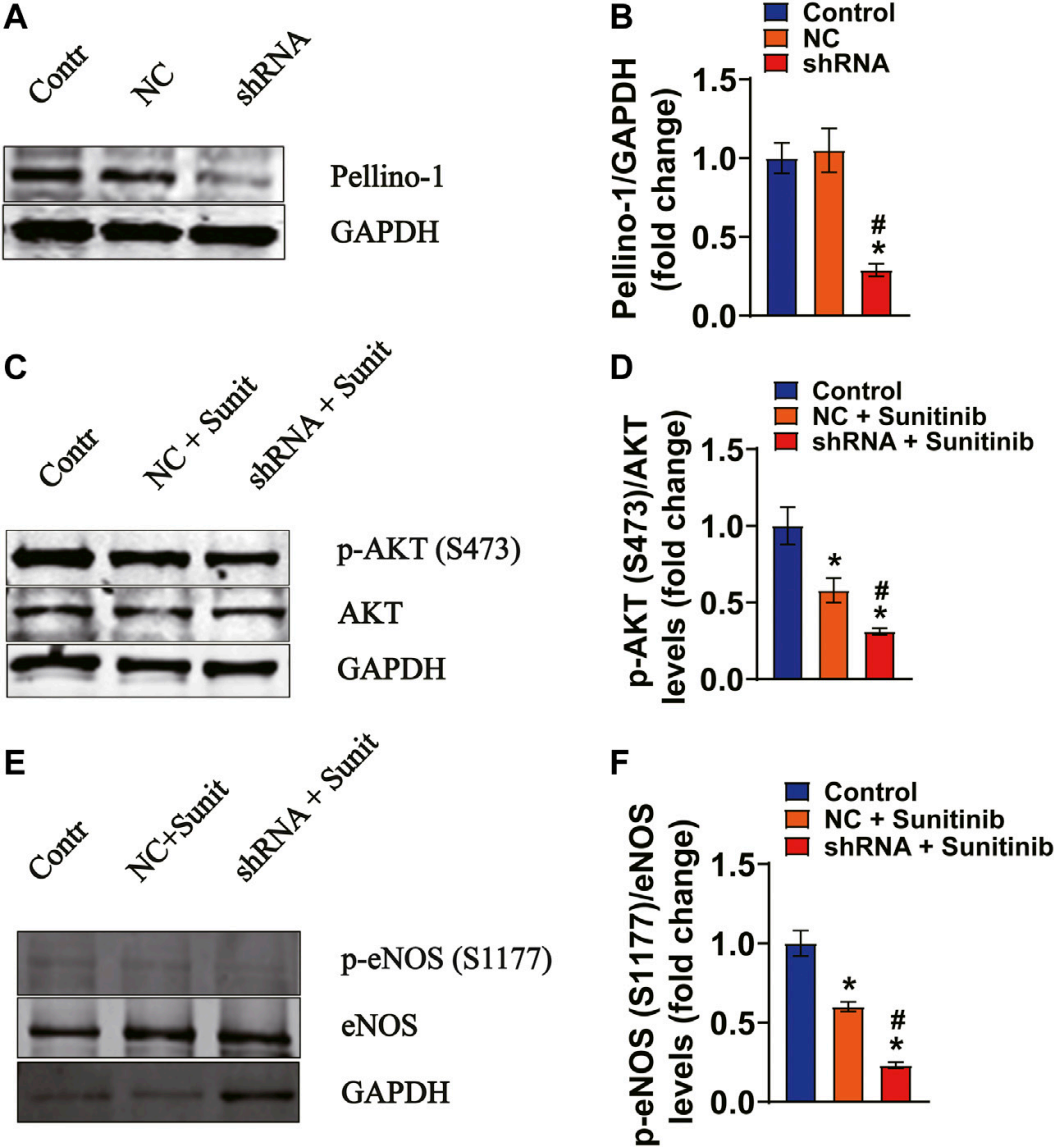
Gene silencing of Pellino-1 exacerbates sunitinib-induced attenuation of AKT and eNOS phosphorylation in MAECs. **(A,B)** Representative western blot images and bar graph demonstrating that knocking down the efficiency of Pellino-1 by the lentivirus-packed rat shRNAs (^*^ *p* < 0.05 vs. control; ^#^ *p* < 0.05 vs. scramble control; *n* = 6). **(C)** Representative western blot images demonstrating the expression of total AKT and phospho-AKT Ser 473 protein performed under each indicated conditions. **(D)** Bar graph representing that sunitinib-induced inhibition of phospho-Ser473 AKT was exacerbated by knocking of Pellino-1 (^*^ *p* < 0.05 vs. control; ^#^ *p* < 0.05 vs. Sunitinib; *n* = 6). **(E)** Representative western blot images representing the expression of total eNOS and phospho-Ser1177 eNOS protein generated under each indicated conditions. **(F)** Statistic bar graph demonstrating that sunitinib-induced decrease in the expression levels of phospho-Ser1177 was further declined by gene silencing of Pellino-1 (^*^ *p* < 0.05 vs. control; ^#^ *p* < 0.05 vs. Sunitinib; *n* = 6). NC representing scramble shRNA transfected group; Sunit indicates sunitinib.

To demonstrate whether there is a direct link between Pellino-1 and AKT/eNOS signaling, MAECs were respectively transfected with either shRNAs against Pellino-1 or scramble shRNA. Our data show that the expression levels of Pellino-1 were dramatically blunted by shRNA, as determined by western blot assays (Figures 7A,B). Moreover, the data show that sunitinib-induced reduction of phosphorylation levels of AKT (Figures 7C,D) and eNOS (Figures 7E,F) were further declined by knocking down of Pellino-1 in MAECs. These results suggest that in addition to insulin resistance-mediated inhibition of vasodilator signaling, a reduced Pellino-1 expression, due to inhibition of VEGF signaling pathway by sunitinib, may also involve in attenuation of AKT/eNOS activation.

## Discussion

The major findings of the present study are: sunitinib administration led to an impairment of vascular function and hypertension in rats, via IRS-1- and Pellino-1-mediated inhibition of AKT/eNOS signaling and reduction of NO production.

Endothelial dysfunction is one of the features of insulin resistance. Pathological stimuli-induced attenuation of endothelial IRS-1/PI3K/AKT/eNOS activation is an important molecular mechanism of endothelial damage, which promotes the incidence of hypertension (Zeng et al., 2000; Perticone et al., 2001; Sironi et al., 2008). The early step of the insulin signaling activation involves in phosphorylation of IRS-1. IRS-1 responses to insulin and insulin-like growth factor (IGF) stimuli via phosphorylation of IRS-1 at tyrosine (Tyr 612) site in endothelial cells (Velloso et al., 1998). In contrast, phosphorylation of IRS-1 at serine sites negatively regulates tyrosine phosphorylation cascade reaction, thereby degrading insulin signaling (Zick, 2001). Sunitinib, a small molecule inhibitor, targets tyrosine kinase as its primary pharmacological action. It has been reported that sunitinib impairs endothelial function by reducing endothelial NO release in both rats and human (Izzedine et al., 2009; Thijs et al., 2015). Moreover, high concentration of sunitinib also impairs endothelium-dependent vasodilation in rats, but not in human with 1 week application of sunitinib (Thijs et al., 2015). The evidence from several clinical trials suggest that NO donors could successfully treat sunitinib-induced hypertension in patients suffering from solid tumor (Dirix et al., 2007; León-Mateos et al., 2015), which further support the notion that the reduced production of NO is an important element in sunitinib-induced hypertension. However, whether sunitinib causes endothelial dysfunction and hypertension via IRS-1/PI3K/AKT/eNOS pathway remains to be demonstrated.

Our results demonstrated the potential molecular mechanisms by which sunitinib causes endothelium-dependent vasodilation dysfunction and hypertension, where we demonstrated the upstream signaling of AKT/eNOS/NO appears to be IRS-1. Our data show that application of sunitinib attenuates phosphorylation of IRS-1 at residue Tyr 612, which is tightly associated with the decreased activities of AKT and eNOS both in the rat mesenteric arteries and in MAECs. Moreover, sunitinib-induced decrease in phosphorylation levels of IRS-1 at residue Tyr 612 may contribute to sunitinib-induced attenuation of insulin signaling, as sunitinib impaired ITT and GTT. Moreover, application of exogenous insulin to stimulate IRS-1 did not affect sunitinib-induced inhibition of IRS-1/AKT/eNOS signaling, suggesting sunitinib, as a TKI, may directly target the IRS-1 to dephosphorylate the residue Tyr 612. It is noteworthy that a decreased expression of total eNOS was observed after sunitinib administration in our *in vivo* experiments. This phenomenon was reported both in the rats model and human specimen that were treated with sunitinib (Eechoute et al., 2012; Santana-Garrido et al., 2020). The possible explanation could be that sunitinib inhibits multiple growth factor signaling, as VEGF promotes eNOS expression (Yang et al., 2012).

It is known that reduction in vessel densities due to inhibition of vascular endothelial growth factor (VEGF) receptor 2/Flk-1 contributes to VEGF inhibitor-induced hypertension. Similar to insulin signaling, VEGF signaling–mediated angiogenesis is depend upon the action of AKT and eNOS (León-Mateos et al., 2015). However, the intermediate molecules that link VEGF receptor and AKT is largely unknown. A recent study revealed that Pellino-1 is an important angiogenic molecule under the control of VEGFR2/Flk-1, as evident in that down regulated Pellino-1 expression during myocardial infarction contributes to suppression of VEGF-mediated angiogenesis via Pellino-1/AKT/eNOS pathway (Thirunavukkarasu et al., 2018). In our experimental model, we found that application of sunitinib led to a significant decrease in the expression levels of Pellino-1 both in the rat artery tissue and in the primary cultured MAECs, suggesting that Pellino-1 may associate with sunitinib-induced decrease in ATK and eNOS activities. This notion was confirmed by the experiments, where sunitinib-induced attenuation of AKT/eNOS activity was further reduced by knocking down the expression of Pellino-1 in the primary cultured MAECs.

Furthermore, we found that the levels of blood glucose were significantly decreased by sunitinib in rats. It's not a common event under insulin resistance condition, as insulin resistance is often accompanied by hyperglycemia (DeFronzo and Ferrannini, 1991; Laakso and Kuusisto, 2014). Billemont and colleagues proposed several potential mechanisms for TKI-induced hypoglycemia including capillary regression of pancreatic islets, IGF-1 modulation, and a reduction of glucose uptake in the context of concomitant gastrointestinal toxicity. (Billemont et al., 2008). Gastrointestinal adverse reactions occur in 53% patients among the patients treated with sunitinib (Motzer et al., 2007; Bhojani et al., 2008). Similar to previously reported results (Steeghs et al., 2008; French et al., 2010), we found that sunitinib administration led to a significant reduction in autonomic food intake and body weight gain. These results suggest that insufficient glucose intake caused by gastrointestinal reaction may be the direct cause of hypoglycemia. In addition, we found that application of sunitinib also resulted in an impairment of insulin sensitivity as determined by ITT and GTT assays and an increase in the levels of serum IAA. Therefore, we considered that sunitinib-induced hypoglycemia is associated with a decrease in glucose intake due to gastrointestinal toxicity, hyperinsulinemia and generation of IAA (Church et al., 2018).

Sunitinib affects insulin clearance in metastatic renal cell carcinoma patients (Thijs et al., 2016). Further study demonstrated that sunitinib directly enhanced glucose-induced insulin secretion (GIIS) in a concentration-dependent manner; the effect of sunitinib on GIIS was further augmented by elevated cAMP levels and the agonists of free fatty acid receptor one (Lutz et al., 2017). Referring to the results obtained from these studies, we argue that sunitinib increases the levels of serum insulin by affecting both insulin secretion and clearance.

Consistence with the results obtained from clinical studies (Thijs et al., 2016), our data suggest that sunitinib at 27.5 mg/kg/day leads to a decrease in insulin sensitivity. However, the results generated from the rats treated with low dose of sunitinib (1.5 or 2.5 mg/kg/day) showed an improved glucose tolerance (Mukai et al., 2014). These different outcomes could suggest that the effects of sunitinib on insulin sensitivity might be dose-dependent. We argue the pathophysiological significance of the results generated from low dose sunitinib, because 1.5–2.5 mg/kg/day is way lower than the concentration of sunitinib used to treat the cancer patients.

We found that sunitinib also significantly elevated circulating cAMP and FFA levels in rats. Increased cAMP may result in FFA release from adipose tissue by activating hormone sensitive lipase to a certain extent; lipid liberation would compensate the energy deficiency due to hypoglycemia, thus playing a protective role against hypoglycemia in the short-term (Doseyici et al., 2014; Winhofer et al., 2015). However, long-term persistence of high levels of FFA is most likely to promote insulin resistance by inhibiting tyrosine phosphorylation of insulin receptor and IRS-1 (Swislocki and Tsuzuki, 1993). Luo and colleagues found that AKT/eNOS signaling pathways are involved in endothelial protection against high-fat diet-induced atherosclerosis in ApoE^−/−^ mice (Luo et al., 2019). Therefore, regulating AKT/eNOS signaling pathways may be profitable for relieving insulin resistance, glyeolipid metabolism disorders, and even endothelial dysfunction caused by sunitinib. However, the mechanisms by which sunitinib leads to increase in the cAMP levels need to be further determined in future studies.

In conclusion, we suggest that sunitinib leads to vascular dysfunction and hypertension via the mechanisms including insulin resistance- and VEGF/Pellino-1-mediated decrease in AKT/eNOS/NO signaling.

## Data Availability

The data that support the findings of this study are available from the corresponding author upon reasonable request.
